# Cell-free circulating mitochondrial DNA content and risk of hepatocellular carcinoma in patients with chronic HBV infection

**DOI:** 10.1038/srep23992

**Published:** 2016-04-11

**Authors:** Ling Li, Hie-Won Hann, Shaogui Wan, Richard S. Hann, Chun Wang, Yinzhi Lai, Xishan Ye, Alison Evans, Ronald E. Myers, Zhong Ye, Bingshan Li, Jinliang Xing, Hushan Yang

**Affiliations:** 1 Division of Population Science, Department of Medical Oncology, Thomas Jefferson University, Philadelphia, PA 19107, USA; 2 Liver Disease Prevention Center, Division of Gastroenterology and Hepatology, Department of Medicine, Thomas Jefferson University, Philadelphia, PA 19107, USA; 3 Institute of Pharmacy, Pharmaceutical College, Henan University, Kaifeng, Henan 475004, China; 4 Department of Environmental Health, School of Public Health, Nantong University, Nantong, Jiangsu 226000, China; 5 Center for Human Genetics Research, Department of Molecular Physiology & Biophysics, Vanderbilt University, Nashville, TN 37232, USA; 6 Experimental Teaching Center, School of Basic Medicine, Fourth Military Medical University, Xi’an, 710032, China

## Abstract

Recent studies have demonstrated a potential link between circulating cell-free mitochondrial DNA (mtDNA) content and cancers. However, there is no study evaluating the association between circulating mtDNA as a non-invasive marker of hepatocellular carcinoma (HCC) risk. We conducted a nested case-control study to determine circulating mtDNA content in serum samples from 116 HBV-related HCC cases and 232 frequency-matched cancer-free HBV controls, and evaluate the retrospective association between mtDNA content and HCC risk using logistic regression and their temporal relationship using a mixed effects model. HCC cases had significantly lower circulating mtDNA content than controls (1.06 versus 2.47, *P* = 1.7 × 10^−5^). Compared to HBV patients with higher mtDNA content, those with lower mtDNA content had a significantly increased risk of HCC with an odds ratio (OR) of 2.19 (95% confidence interval [CI] 1.28–3.72, *P* = 0.004). Quartile analyses revealed a significant dose-dependent effect (*P*_trend_ = 0.001) for this association. In a pilot longitudinal sub-cohort of 14 matched cases-control pairs, we observed a trend of dramatically decreased mtDNA content in cases and slightly decreased mtDNA content in controls, with a significant interaction of case-control status with time (*P*_interaction_ = 0.049). Our findings suggest that circulating mtDNA is a potential novel non-invasive biomarker of HCC risk in HBV patients.

Hepatocellular carcinoma (HCC) is a leading cause of cancer death, and chronic hepatitis B virus (HBV) infection represents the most common etiology of HCC worldwide^1^. In the United States, although hepatitis C virus (HCV) infection and alcohol and fat liver diseases are more common HCC etiological factors of HCC, HBV-related HCC has also grown significantly due to the influx of immigrants from HBV endemic regions such as Asia. It was reported that there were about 1.5 million chronic HBV-infected patients among the more than 40 million Americans born outside of the United States^2^. Moreover, over 60% of these HBV patients are relatively young, which may significantly increase the HBV-induced HCC risk and associated medical costs in the next a few decades^3^. These facts highlight the importance of risk prediction, close surveillance, and early diagnosis in HCC management.

Mitochondria are organelles in cytoplasm of the eukaryotic cells that play an important role in essential cellular functions such as energy metabolism, free radical generation, calcium homeostasis, and apoptosis^4^. Each cell contains several hundred to thousand mitochondria and each mitochondrion possesses its own genome^5^. Mitochondrial DNA (mtDNA) consists of an approximately 16.5 kb circular, maternally inherited, double-stranded extrachromosomal DNA^6^. Unlike nuclear DNAs, mtDNA does not have protective histones and sophisticated DNA repair mechanisms, which render it extremely susceptibility to oxidative stress and other genotoxic insults^7^. Moreover, high levels of reactive oxygen species (ROS) are generated around mtDNAs during metabolic events occurring in mitochondria. This environment of high oxidative stress also contributes to the high susceptibility of mtDNA to mutagenesis. As a defense mechanism, mitochondria increase their DNA copy numbers to mitigate the negative effects resulting from the high rates of mutations in their genomes^8^. The abnormal alteration of mtDNA copy number is well documented for numerous malignancies, including HCC^9^,^10^,^11^,^12^.

Biomarkers based on circulating DNA samples have unique advantages over other specimens due to their non-invasive nature as well as the capability of repetitive sampling^13^,^14^,^15^. Recently, it has been reported that the mtDNA content from plasma or serum samples are significantly associated with various diseases, such as Ewing’s sarcoma^16^, trauma^17^, and microbial infection^18^,^19^. It has also been associated with the mortality of patients in intensive care units^20^. However, as yet there is no reported study evaluating the association between circulating mtDNA content and HCC risk. In the current study, we sought to evaluate the association between circulating mtDNA content and the risk of HCC in patients with chronic HBV infection.

## Results

### Characteristics of study population

The patients’ characteristics were summarized in Table 1. This analysis included 116 HBV-related HCC cases and 232 cancer-free HBV controls that were 1:2 frequency-matched on age (mean ± standard deviation [SD], 55.7 ± 9.1 years *vs*. 54.1 ± 8.8 years, *P* = 0.123) and gender (*P* = 1.000). Additionally, no significant difference was observed in other major host characteristics, such as smoking status (*P* = 0.491), drinking status (*P* = 0.494), and family history of cancer (*P* = 0.742). As expected, a significantly higher percentage of cirrhotic patients was noted in HBV-related HCC cases (77.6%) than cancer-free HBV controls (39.7%) (*P* = 2.4 × 10^−11^).

### Distributions of circulating mtDNA content in cases and controls by patients’ characteristics

The overall and stratified distributions of circulating mtDNA content between cases and controls were shown in Table 2. Overall, cases had significantly lower mtDNA content than controls (median, 1.06 [quartile range, 0.23–3.09] *vs*. 2.47 [0.90–5.15], *P* = 1.7 × 10^−5^). In stratified analyses, the significantly lower mtDNA content in cases was evident in all strata. In addition, we compared mtDNA differences between the strata of each variable in cases and controls separately. We observed a significantly lower mtDNA content in older than younger patients, and in males than females (*P* = 1.1 × 10^−6^ and 1.2 × 10^−5^, respectively) in controls. There was no significant difference between the strata of other variables in controls and all variables in cases. We further compared mtDNA content in the patients with different status of liver diseases by analyzing mtDNA content in three groups of patients: cancer-free HBV patients without liver cirrhosis, cancer-free HBV patients with liver cirrhosis, and HBV patients who were diagnosed as HCC. We found no statistically significant differences in the distributions of major patient characteristics among the three groups (Supp. Table 1). The median value (quartile range) of mtDNA content was 2.58 (1.12–5.21) in 140 HBV patients without cirrhosis, 2.21 (0.55–4.94) in 92 cirrhotic HBV patients, and 1.06 (0.23–3.09) in 116 HBV-HCC patients (*P* < 0.0001), showing an apparent decreasing trend in mtDNA content from non-cirrhotic HBV patients, to cirrhotic HBV patients, and then to HCC patients.

### Association between circulating mtDNA content and HCC risk

The association between circulating mtDNA content and HBV-related HCC risk was estimated using unconditional logistic regression model by analyzing the mtDNA content as a categorical variable based on the cutoff of median or quartile distribution of mtDNA content in controls. As shown in Table 3, patients with a lower level of serum mtDNA content (≤2.47) exhibited a significantly increased HCC risk with a crude OR of 2.22 (95% CI 1.39–3.56, *P* = 8.7 × 10^−4^) in univariate analysis and an adjusted OR of 2.19 (1.28–3.72, *P* = 0.004) in multivariate analysis adjusting for age, gender, smoking status, drinking status, family history of cancer, and cirrhosis, compared to those with a higher mtDNA content (>2.47). In quartile analysis, using the patients with the highest level of mtDNA content as reference, patients with lower levels of mtDNA content showed significantly increased HCC risk in a dose-dependent manner in both univariate and multivariate analyses (*P*_for trend_ = 1.6 × 10^−4^, and 0.001, respectively). To analyze the confounding effect of alpha fetoprotein (AFP), the most commonly used HCC marker in clinics, we conducted a subset analysis in 252 patients with available AFP data. The results of the subset analysis showed that the association between mtDNA content and HCC risk remained significant (*P*_for trend_ = 0.001) after adjusting for covariates including AFP (Table 3). Similar results were also noticed when major liver enzymes (e.g., alkaline phosphatase [ALP], aspartate aminotransferase [AST], alanine aminotransferase [ALT], gamma-glutamyltransferase [GGT]) were added to the multivariate analyses (Supp. Table 2). Moreover, we did not identify any correlation between mtDNA content and AFP level or tumor size (data not shown).

### Association between circulating mtDNA content and HCC risk stratified by patients’ characteristics

We further evaluated the association between circulating mtDNA content and HCC risk stratified by patients’ characteristics using multivariate analysis adjusted for age, gender, smoking status, drinking status, family history of cancer, and cirrhosis, where appropriate. We found that the significantly increased HCC risk conferred by decreased mtDNA content was evident in younger patients with an adjusted OR of 3.82 (1.69–8.60, *P* = 0.001) but not in older patients (*P* = 0.682) (Table 4). Moreover, a borderline statistically significant interaction effect was noted between age and mtDNA content on the risk of HBV-HCC (*P*_for interaction_ = 0.062). An increased HCC risk associated with lower mtDNA content was also observed in both females (*P* = 0.022) and males (*P* = 0.044), never smokers (*P* = 0.011), never drinkers (*P* = 0.013), patients with a family history of cancer (*P* = 0.014), and patients with cirrhosis (*P* = 0.043). However, none of these variables exhibited a significant interaction with mtDNA content, except gender for which a borderline significant interaction was noted (*P*_for interaction_ = 0.087) (Table 4).

### Diagnostic value of mtDNA in combination with common demographic and clinical variables

We further explored the diagnostic value of mtDNA in both the overall dataset and the subsets of patients with available AFP or liver enzyme data. First, we found that in the overall dataset, when mtDNA content was added to multivariate analysis adjusting for age, gender, smoking status, drinking status, family history of cancer, and cirrhosis, the area under the curve (AUC) of receiver operating characteristic (ROC) curve significantly increased from 0.7133 (0.6582–0.7683) to 0.7511 (0.6981–0.8041) (*P* = 0.046) (Supp. Fig. 1A), indicating that mtDNA provided additional diagnostic value to the common demographic variables. Second, we conducted analyses in the subset of patients with available clinical values to compare the diagnostic performance of mtDNA with AFP or liver enzymes. There was no statistically significant difference in the AUCs between the model including mtDNA content and that including AFP (*P* = 0.527) or each of the liver enzyme (*P* = 0.149 for ALP, 0.216 for AST, 0.135 for ALT, and 0.545 for GGT) (Supp. Fig. 1B). Third, to investigate whether mtDNA content provided additional diagnostic value over AFP, we further compared the AUCs derived from the multivariate model including demographic variables plus AFP, and that including demographic variables plus both AFP and mtDNA. The AUC was 0.8020 (0.7400–0.8641) in the multivariate model including demographic variables plus AFP, and the AUC significantly increased to 0.8498 (0.7964–0.9032) after adding mtDNA to the model (*P* = 0.032, Supp. Fig. 2A). Similar results were found in the models including each of the liver enzymes (Supp. Fig. 2B–E). These new data demonstrated that mtDNA provided additional diagnostic value when jointly used with AFP or common liver enzymes.

### Pilot longitudinal analysis

**Figure 1:**
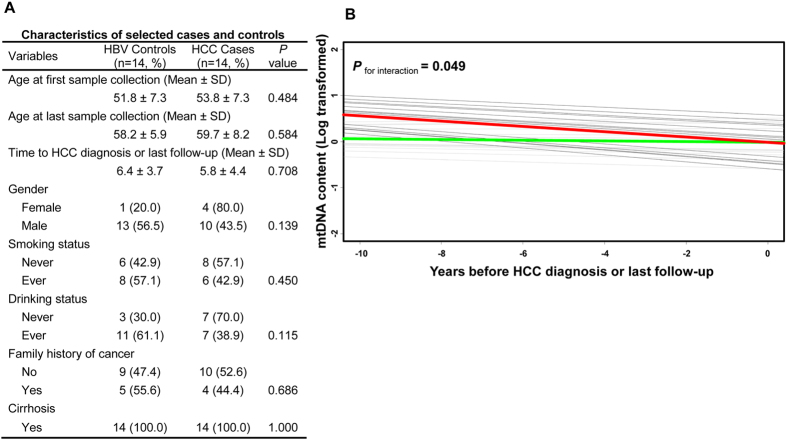
Pilot longitudinal analysis of circulating mtDNA content and the risk of HBV-related HCC. (**A**) Characteristics of 14 HBV-related HCC cases and 14 frequency-matched cancer-free HBV controls for longitudinal analysis. (**B**) Mixed effects model was used to analyze circulating mtDNA content and HCC risk through an interaction of case-control status with time in 14 case-control pairs. Grey lines, individual HBV patient who remained cancer-free (controls); black lines, individual HBV patient who developed HCC (cases); green lines, overall trend of mtDNA change in controls; red lines, overall trend of mtDNA change in cases.

In order to illuminate the dynamic change of circulating mtDNA over time, we conducted a pilot longitudinal analysis in 14 pairs of cases and controls identified from the patients included in this analysis that had at least three samples available before HCC diagnosis (for cases) or last follow-up (for controls). Cases in this pilot analysis were those cancer-free HBV patients who developed HCC after at least one year of follow up. Controls were those HBV patients who remained cancer-free at last follow-up. The cases and controls were adequately matched on age at first sample collection (*P* = 0.484), age at last sample collection (*P* = 0.584), follow-up time (*P* = 0.708), gender (*P* = 0.139), smoking status (*P* = 0.450), drinking status (*P* = 0.115), and family history of cancer (*P* = 0.686). All cases and controls had cirrhosis at first sample collection (Fig. 1A). We measured mtDNA content in all available serum samples after study entry until HCC diagnosis (for cases) or last follow-up (for controls). Using a mixed effects model, we observed a trend of dramatically decreased mtDNA content in cases and a slightly decreased mtDNA content in controls during follow-up, with a significant interaction of case-control status with time (*P*_for interaction_ = 0.049) (Fig. 1B).

## Discussion

In the present study, we evaluated the association between circulating mtDNA from cell-free serum samples and HCC risk in a clinic-based cohort of HBV-infected patients. We found that a lower level of serum mtDNA content was significantly associated with an increased risk of HBV-related HCC in a dose-dependent manner, suggesting circulating mtDNA may be a novel non-invasive marker of HCC risk.

mtDNA exhibits about 10- to 200-fold higher rate of mutagenesis than that of nuclear DNA under an oxidative stress environment, due to the lack of protective histone proteins and the limited capacity of mitochondrial DNA repair system^7^,^21^. A growing body of evidence has described the alterations in the copy number of mtDNA in a cancer-specific manner^9^,^10^,^11^,^12^. The increase of mtDNA copy number, partially resulting from enhanced oxidative stress and feedback response that compensates for mitochondria with impaired respiratory function or elevated mtDNA mutations, has been found in a variety of malignancies, such as colorectal, esophageal, and lung cancers^22^,^23^,^24^,^25^. In comparison, studies in malignancies such as breast cancer, gastric cancer, renal cell carcinoma, Ewing’s sarcoma, and HCC reported a decreased mtDNA copy number^26^,^27^,^28^,^29^,^30^. It was proposed that point mutations adjacent to the replication region in the D-loop, the non-coding region regulating both replication and transcription of mitochondrial genome, may play an important role in the reduction of mtDNA copy number in HCC, breast cancer and Ewing’s sarcoma^9^,^26^,^30^. Genetic and genomic alterations in key players such as *TP53* and mtDNA polymerases have also been linked to the decreased mtDNA copy number in colorectal and breast cancers^31^,^32^. In HCC, increased expression of nuclear DNA-encoded transacting factor, mitochondrial single strand DNA binding protein, which is important to mitochondrial biogenesis and mtDNA maintenance, was also associated with decreased copy number of mtDNA^10^. In the current study, we found that a lower level of mtDNA content in a serum sample was significantly associated with an increased HCC risk, which was consist with the findings in other HBV-HCC studies using tissues or peripheral blood leukocyte^10^,^29^,^33^. Injury to the respiratory machinery was considered a critical event in tumorigenesis resulting from mtDNA dysfunction^34^. The decrease in mtDNA content could decrease oxidative phosphorylation capacity and enhance production of ROS in aerobic metabolism, which is related to DNA damage, fast cellular growth, apoptosis resistance, and malignant cellular transformation^33^. Functional characterizations are needed to further elucidate the mechanisms underlying the association.

Although the origins of circulating DNAs in cell-free samples such as serum or plasma are not fully clear, it is commonly recognized that the majority of these nucleic acids are derived from necrotic and apoptotic cells from circulation system or target tissues^35^,^36^. This is especially relevant for HBV patients, in whom chronic inflammation further contributes to the generation and release of circulating nucleic acids through promoting repetitive liver damage^37^,^38^,^39^. Circulating DNAs have the unique advantage to serve as “liquid biopsy”, which may be used as cost-effective and non-invasive surrogates for tissue biopsies in cancer risk prediction and early detection^40^. Detection of aberrant changes of circulating mtDNA is becoming an increasingly important tool to assist early cancer diagnosis with some unique advantages over nuclear DNA^15^,^41^,^42^. For instance, the much shorter and more simply organized mitochondrial genome makes screening much easier and more cost-effective than for the nuclear genome. In addition, the higher number of mtDNA copy makes it easier to detect mtDNA content than nuclear DNAs in body fluid samples such as plasma and serum, in which the total DNA concentration is at very low abundance. Previous studies using peripheral blood leukocyte or tissue samples have shown an significant association between a lower mtDNA copy number variations and an elevated HCC risk in HBV patients^29^,^33^. Our study confirmed these findings and takes a step further to demonstrate that circulating mtDNA content in cell-free samples may be used as a novel cost-effective and non-invasive marker of HCC risk in HBV patients.

The concentration and components of circulating cell-free DNA vary in a wide range that is affected by physiological and pathological status of individuals^14^. Therefore, a single baseline circulating mtDNA measurement may not be reliable enough to yield robust findings. To help address this concern, we conducted a pilot longitudinal case-control analysis by measuring mtDNA content in all of the available serum samples collected before HCC diagnosis or last follow-up for each patient. In this analysis, we observed a slightly decreasing trend of mtDNA content along with increased age in HBV patients who did not develop HCC, which was not surprising because a negative correlation between mtDNA content and age has been documented^43^. Interestingly, a significant trend of reduction in mtDNA content was noticed in HBV patients who developed into HCC during follow up, consistent with the results of the main effects analysis of our study. Moreover, a significant interaction of case-control status with time was identified. These findings of the pilot longitudinal analysis established a temporal link and further substantiated the association between reduced circulating mtDNA content and increased HCC risk. However, due to the unplanned nature and small sample size, the results of the pilot longitudinal analysis need to be interpreted with caution and validated in future larger and well-designed longitudinal cohorts.

A major strength of our study is the unique and highly homogenous HBV patient population. Our study population is restricted to Korean Americans with HBV-infected patients only, which eliminates the potential confounding effects conferred by ethnicity and disease etiology. The strict matching criteria between cases and controls further minimized the confounding from other major risk factors. The non-invasive nature of measuring circulating mtDNA derived makes it possible for repetitive patient monitoring during follow-up and treatment. Furthermore, our findings were greatly strengthened by the pilot longitudinal analysis based on our unique serial collection of serum samples. There are also some limitations in our study. First, the restriction of the analysis to Korean HBV patients, although increasing study homogeneity, limited its generalizability. Second, we were not able to compare mtDNA content in serum with that in liver tissues (either HCC or normal tissue) due to unavailability of tissue samples in our study. Third, because the clinical data in our study were obtained through retrospective chart review, we were not able to conduct the multivariate analyses adjusting for all of the clinical variables in the same model due to the missing values of these variables. We also could not evaluate the correlations between mtDNA content and other clinicopathological variables such as tumor stage or vascular invasion, because these data were not available in our medical charts. In addition, although we detected a higher diagnostic sensitivity of Magnetic Resonance Imaging (MRI) compared to mtDNA alone (75.7% from multiple MRI examinations vs. 69.0% from single mtDNA measurement), the lack of MRI data in many of cancer-free HBV patients in our study made it impossible for us to compare the specificities of mtDNA with that of MRI. Last, owing to the retrospective case-control design, our study is also limited by the reverse-causation issue that is inherent in most retrospective studies, and cannot differentiate the causal relationship between circulating mtDNA content and HCC development. Further large-scale prospective studies are warranted to confirm our findings.

In conclusion, our study reports the first epidemiological evidence that lower circulating mtDNA content in cell-free serum samples is significantly associated with an increased risk of HCC in HBV patients. If validated, circulating mtDNA content may serve as a promising non-invasive biomarker for HCC diagnosis.

## Methods

### Study population

The subjects of this study were identified from an existing and ongoing clinic-based patient cohort. Patients were consecutively enrolled when they visited the Liver Disease Prevention Center at the Division of Gastroenterology and Hepatology in Thomas Jefferson University Hospital for the treatment of liver diseases such as HBV infection, HCV infection, cirrhosis, and HCC. Patient enrollment started in 1988 and is still ongoing. More details regarding this cohort were as previously described^3^. Since more than 90% of the patients in this cohort were of Korean ancestry and had HBV infection, to minimize the potential confounding effects from ethnicity and disease etiology, we restricted the study subjects in the current analysis to Korean HBV patients only. This study was approved by the Institutional Review Board of Thomas Jefferson University and the methods were carried out in accordance with the approved guidelines. A written informed consent was obtained from each patient.

### Epidemiologic and clinical data collection

Demographic and clinical data were obtained for each patient through medical chart review and/or consulting with the treating physicians. Demographic variables collected in this study included age, gender, ethnicity, smoking status, drinking status, family history of cancer, and cirrhosis status. Individuals who had never smoked or had smoked less than 100 cigarettes in his or her lifetime were defined as a never smoker; otherwise as an ever smoker. Never drinker was defined as those who never consumed alcohol or consumed less than one drink per month; otherwise as an ever drinker. Liver cirrhosis and HCC were diagnosed by the combined use of clinical tests such as platelet counts and Child–Pugh scores and imaging studies especially magnetic resonance imaging, complemented by serum levels of AFP. Blood samples were drawn from patients when taking the clinical laboratory testing, and the extracted serum samples were stored at −80 °C for research purposes. The blood samples from most HCC patients were collected at the time of HCC diagnosis. For a few patients who had no blood samples collected at HCC diagnosis, we used the samples with collection dates close to the time of HCC diagnosis.

### Measurement of serum mtDNA content

First, circulating DNA was isolated from 200 μL serum sample using QIAamp DNA Blood Mini kit (Qiagen, Carlsbad, CA) according to the manufacturer’s protocol. The relative mtDNA content was measured by quantitative real-time polymerase chain reaction (qRT-PCR) using a modified protocol described by previous studies^28^,^33^, in which the ratio of the copy number for mitochondrial *ND1* gene to the copy of a human single copy gene *36B4* was used to determine the relative mtDNA content. Briefly, the 6 μL of qRT-PCR reaction for both *ND1* and *36B4* genes consists of 1 X SYBR Green Master Mix (Applied Biosystems, CA, USA), 200 nmol/L each primer, and 2 μL of purified serum DNA sample. The primer sequences were as follows: Forward *ND1* primer: 5′-CCCTAAAACCCGCCACATCT-3′; Reverse *ND1* primer: 5′-GAGCGATGGTGAGAGCTAAGGT-3′; Forward *36B4* primer: 5′-CAGCAAGTGGGAAGGTGTAATCC-3′; Reverse *36B4* primer: 5′-CCCATTCTATCATCAACGGGTACAA-3′. The thermal cycling conditions for both genes were pre-denatured at 95 °C for 10 min, followed by 40 cycles of 95 °C for 15 seconds and 58 °C for 1 minute with data collection. All samples were assayed in duplicate on a 384-well plate using ViiA 7 qRT-PCR system (Applied Biosystems, CA). The same negative control and calibrator DNA samples were incorporated into each plate for quality control and calibration of PCR efficiency. A reference DNA sample was used to construct a standard curve for mtDNA measurement in each palate. For each standard curve, the reference DNA sample was serially diluted by 1:2 to produce a ten-point standard curve with the initial concentration at 20 ng/μL. The *R*^*2*^ for each standard curve was ≥0.98, with acceptable standard deviation set at 0.25 for the cycle threshold (Ct) values. If the Ct value of a tested sample was out of the range of standard curve, the sample was repeated.

### Statistical analyses

Statistical analyses were performed using the SAS software version 9.2 (SAS Institute, Cary, NC) or STATA 12.0 package (STATA Corp, College Station, TX). The differences in the distributions of patients’ characteristics between cases and controls were compared using Chi-square test for categorical variables, and student’s *t* test for continuous variables. The differences of serum mtDNA content between cases and controls or between dichotomized patients’ characteristics were compared by Wilcoxon rank-sum test. Serum mtDNA was analyzed as categorical variable by cutoffs at median or quartile values in the controls. The association between mtDNA and HCC risk was estimated by unconditional logistic regression model using univariate and multivariate analyses to determine the odds ratios (ORs) and 95% confidence intervals (95% CI). In subset analyses, AFP or serum liver enzymes were added into the multivariable analysis. Discrimination accuracy for HCC diagnosis was evaluated by AUC of ROC curve. The test for interaction between patients’ characteristics and mtDNA on HCC risk was performed by including a cross-product term into the logistic regression model. In the pilot longitudinal analysis to analyze the temporal relationship between mtDNA content and HCC risk, mtDNA content in 14 case-control pairs (matched on age at first sample collection, age at last sample collection, follow-up time, gender, smoking status, drinking status, family history of cancer, and cirrhosis ) were plotted after log transformation against follow-up time. A mixed effects model was used to analyze mtDNA content through an interaction of the case-control status with time. All statistical tests were two sided, and *P* < 0.05 was considered as the threshold of statistical significance.

## Additional Information

**How to cite this article**: Li, L. *et al.* Cell-free circulating mitochondrial DNA content and risk of hepatocellular carcinoma in patients with chronic HBV infection. *Sci. Rep.* **6**, 23992; doi: 10.1038/srep23992 (2016).

## Supplementary Material

Supplementary Information

## Figures and Tables

**Table 1:** Summary of the study population.

Variables	HBV Controls (n = 232, %)	HCC Cases (n = 116, %)	*P* value
Age (Mean ± SD)	54.1 ± 8.8	55.7 ± 9.1	0.123
Gender			
Female	26 (11.2)	13 (11.2)	
Male	206 (88.8)	103 (88.8)	1.000
Smoking status			
Never	135 (58.2)	63 (54.3)	
Ever	97 (41.8)	53 (45.7)	0.491
Drinking status			
Never	127 (54.7)	59 (50.9)	
Ever	105 (45.3)	57 (49.1)	0.494
Family history of cancer			
No	160 (69.0)	82 (70.7)	
Yes	72 (31.0)	34 (29.3)	0.742
Cirrhosis			
No	140 (60.3)	26 (22.4)	
Yes	92 (39.7)	90 (77.6)	2.4 × 10^−11^

**Table 2:** Distributions of circulating mtDNA content by host characteristics in cases and controls.

Variables	mtDNA: Median (Quartile range)		*P*^†^ value
	HBV controls	HCC cases	
Overall	2.47 (0.90–5.15)	1.06 (0.23–3.09)	1.7 × 10^−5^
Age, y			
≤54.3	3.37 (1.84–5.77)	1.22 (0.30–3.92)	1.5 × 10^−4^
>54.3	1.36 (0.35–3.75)	0.92 (0.23–2.75)	0.049
*P*^‡^	1.1 × 10^−6^	0.287	
Gender			
Female	6.66 (2.76–10.94)	1.20 (0.68–2.57)	0.005
Male	2.18 (0.88–4.43)	1.02 (0.22–3.28)	3.1 × 10^−4^
*P*^‡^	1.2 × 10^−5^	0.403	
Smoking status			
Never	2.57 (0.95–5.17)	0.92 (0.22–3.28)	0.001
Ever	2.17 (0.88–4.75)	1.20 (0.27–2.79)	0.012
*P*^‡^	0.438	0.905	
Drinking status			
Never	2.56 (0.94–5.10)	0.86 (0.19–3.28)	0.001
Ever	2.27 (0.89–5.21)	1.36 (0.29–2.84)	0.011
*P*^‡^	0.995	0.522	
Family history of cancer			
No	2.23 (0.77–4.73)	1.15 (0.27–3.49)	0.005
Yes	2.71 (1.19–5.55)	0.93 (0.18–2.75)	3.2 × 10^−4^
*P*^‡^	0.143	0.509	
Cirrhosis			
No	2.58 (1.12–5.21)	0.68 (0.18–1.95)	0.001
Yes	2.21 (0.55–4.94)	1.21 (0.27–3.49)	0.018
*P*^‡^	0.286	0.335	

*P*^**†**^, comparisons between cases and controls.

*P*^‡^, comparisons between the two strata of each individual variable.

**Table 3:** The association of circulating mtDNA content with HCC risk.

mtDNA	HBVcontrols	HCCcases	Univariate		Multivariate†		Multivariate‡		Multivariate§	
			OR (95% CI)	*P* value	OR (95% CI)	*P* value	OR (95% CI)	*P* value	OR (95% CI)	*P* value
By median										
>2.47	116	36	1.00		1.00		1.00		1.00	
≤2.47	116	80	2.22 (1.39–3.56)	8.7 × 10^−4^	2.19 (1.32–3.63)	0.003	2.19 (1.28–3.72)	0.004	2.95 (1.32–6.58)	0.008
By quartile										
>4.93	58	19	1.00		1.00		1.00		1.00	
4.93–	58	17	0.90 (0.42–1.89)	0.771	0.95 (0.44–2.04)	0.887	0.88 (0.39–1.98)	0.758	2.94 (0.95–9.06)	0.060
2.47–	58	26	1.37 (0.68–2.74)	0.376	1.42 (0.69–2.94)	0.340	1.41 (0.66–3.03)	0.376	3.46 (1.14–10.46)	0.030
≤0.55	58	54	2.84 (1.50–5.37)	0.001	2.96 (1.48–5.93)	0.002	2.77 (1.33–5.76)	0.006	9.03 (2.31–35.23)	0.002
*P*_for trend_				1.6 × 10^−4^		4.8 × 10^−4^		0.001		0.001

^†^Adjusted for age, gender, smoking status, drinking status, and family history of cancer.

^‡^Adjusted for age, gender, smoking status, drinking status, family history of cancer, and cirrhosis.

^§^Adjusted for age, gender, smoking status, drinking status, family history of cancer, cirrhosis, and alpha fetoprotein.

**Table 4:** The associations of circulating mtDNA content with HCC risk stratified by patients’ characteristics.

Variables	mtDNA	HBV controls	HCC cases	OR (95% CI)†	*P* value	*P*_for interaction_
Age						
≤ 54.3	Higher	76	16	1.00		
	Lower	40	31	3.82 (1.69–8.60)	0.001	
> 54.3	Higher	40	20	1.00		
	Lower	76	49	1.16 (0.57–2.36)	0.682	0.062
Gender						
Female	Higher	22	4	1.00		
	Lower	4	9	27.99 (1.61–486.69)	0.022	
Male	Higher	94	32	1.00		
	Lower	112	71	1.78 (1.02–3.10)	0.044	0.087
Smoking status						
Never	Higher	70	19	1.00		
	Lower	65	44	2.60 (1.24–5.43)	0.011	
Ever	Higher	46	17	1.00		
	Lower	51	36	1.82 (0.83–4.02)	0.136	0.414
Drinking status						
Never	Higher	65	17	1.00		
	Lower	62	42	2.68 (1.24–5.80)	0.013	
Ever	Higher	51	19	1.00		
	Lower	54	38	1.87 (0.88–3.96)	0.101	0.575
Family history of cancer						
No	Higher	75	26	1.00		
	Lower	85	56	1.88 (0.99–3.57)	0.053	
Yes	Higher	41	10	1.00		
	Lower	31	24	3.97 (1.32–11.93)	0.014	0.308
Cirrhosis						
No	Higher	73	6	1.00		
	Lower	67	20	2.78 (0.93–8.36)	0.069	
Yes	Higher	43	30	1.00		
	Lower	49	60	1.91 (1.02–3.59)	0.043	0.184

^†^ORs were adjusted for age, gender, smoking status, drinking status, family history of cancer, and cirrhosis, where appropriate.
