# Supplementary material for: Cell-free circulating mitochondrial DNA content and risk of hepatocellular carcinoma in patients with chronic HBV infection
Source: Sci Rep. 2016 Apr 11;6:23992. doi: 10.1038/srep23992 (PMC4827061; doi:10.1038/srep23992)
Supplement: Supplementary Information [file srep23992-s1.pdf]

Cell-free circulating mitochondrial DNA content and risk of hepatocellular carcinoma in patients with chronic HBV infection

Ling Li[1,*], Hie-Won Hann[2,*], Shaogui Wan[1,3,*], Richard S. Hann[2], Chun Wang[1,4], Yinzhi Lai[1], Xishan Ye[1], Alison Evans[1], Ronald E. Myers[1], Zhong Ye[1], Bingshan Li[5], Jinliang Xing[6] & Hushan Yang[1]

Supplementary Table 1. Summary of the study population.

Variables	Cancer-free HBV patients		HCC patients (n=116, %)	*P* value
	Non-cirrhotic (n=140, %)	Cirrhotic (n=92, %)		
**Age (Mean ± SD)**	53.3 ± 9.0	55.4 ± 8.3	55.7 ± 9.1	0.061
**Gender**				
Female	17 (12.1)	9 (9.8)	13 (11.2)	
Male	123 (87.9)	83 (90.2)	103 (88.8)	0.856
**Smoking status**				
Never	83 (59.3)	52 (56.5)	63 (54.3)	
Ever	57 (40.7)	40 (43.5)	53 (45.7)	0.723
**Drinking status**				
Never	81 (57.9)	46 (50.0)	59 (50.9)	
Ever	59 (42.1)	46 (50.0)	57 (49.1)	0.398
**Family history of cancer**				
No	90 (64.3)	70 (76.1)	82 (70.7)	
Yes	50 (35.7)	22 (23.9)	34 (29.3)	0.153

Supplementary Table 2. The associations of circulating mtDNA content with HCC risk.

mtDNA	Multivariate[a]		Multivariate[b]		Multivariate[c]		Multivariate[d]	
	OR (95%CI)	*P* value	OR (95%CI)	*P* value	OR (95%CI)	*P* value	OR (95%CI)	*P* value
**By median**								
> 2.47	1.00		1.00		1.00		1.00	
≤ 2.47	2.45 (1.18-4.29)	**0.010**	2.54 (1.27-5.09)	**0.009**	2.70 (1.39-5.25)	**0.004**	2.69 (1.04-6.98)	**0.040**
**By quartile**								
>4.93	1.00		1.00		1.00		1.00	
4.93-	2.54 (1.02-6.29)	**0.040**	2.67 (1.04-6.87)	**0.040**	2.92 (1.14-7.47)	**0.030**	1.96 (0.52-7.39)	0.320
2.47-	2.82 (1.17-6.77)	**0.020**	3.15 (1.19-8.32)	**0.020**	3.76 (1.51-9.36)	**0.004**	2.37 (0.65-8.66)	0.193
≤0.55	4.12 (1.55-10.95)	**0.004**	5.52 (1.88-16.22)	**0.002**	5.15 (1.84-14.41)	**0.002**	7.41 (1.66-33.12)	**0.009**
$P_{\text{for trend}}$		**0.003**		**0.002**		**0.001**		**0.003**

Multivariate models adjusting for age, gender, smoking status, drinking status, family history of cancer, cirrhosis, and each of the liver enzymes (a. ALP, b. AST, c. ALT, d. GGT).

A

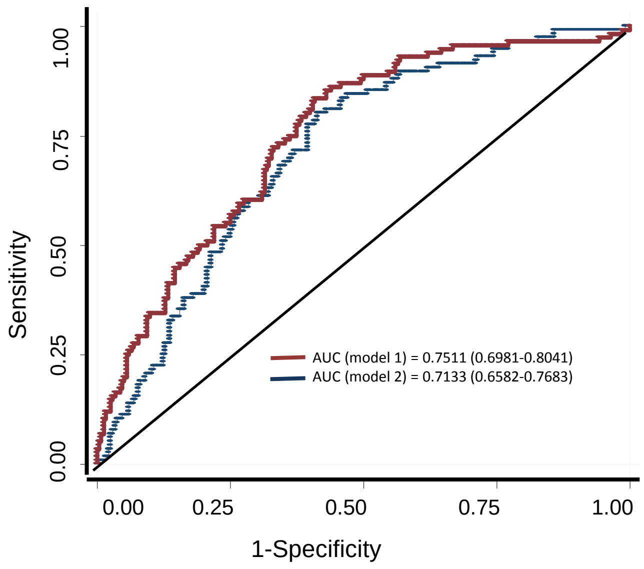

B

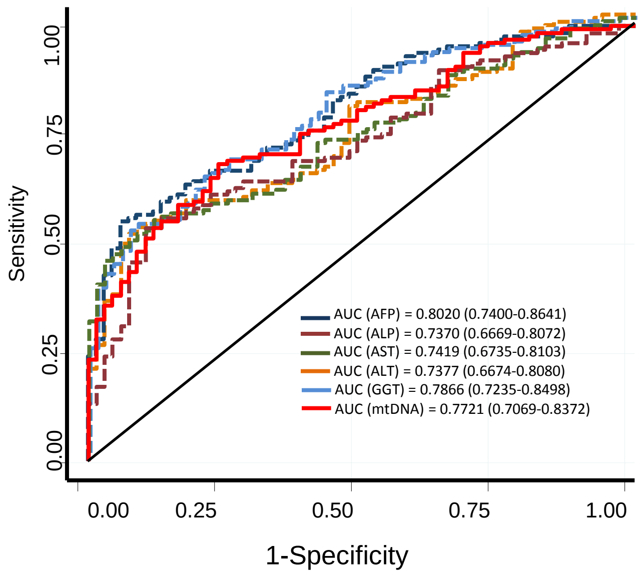

Supplementary Figure 1. Discrimination accuracy for different HCC diagnosis models. Discrimination accuracy for HCC diagnosis was evaluated by AUC of ROC curves. The AUCs were compared (**A**) between the model including age, gender, smoking status, drinking status, family history of cancer, cirrhosis, and mtDNA content (model 1), and the model including age, gender, smoking status, drinking status, family history of cancer, and cirrhosis (model 2) (*P*=0.046) in the overall dataset; (**B**) between the model including age, gender, smoking status, drinking status, family history of cancer, cirrhosis, and mtDNA content; and the models including age, gender, smoking status, drinking status, family history of cancer, cirrhosis, and AFP (*P*=0.527) or each of the liver enzymes including ALP (*P*=0.149), AST (*P*=0.216), ALT (*P*=0.135), and GGT (*P*=0.545), in the subset analyses.

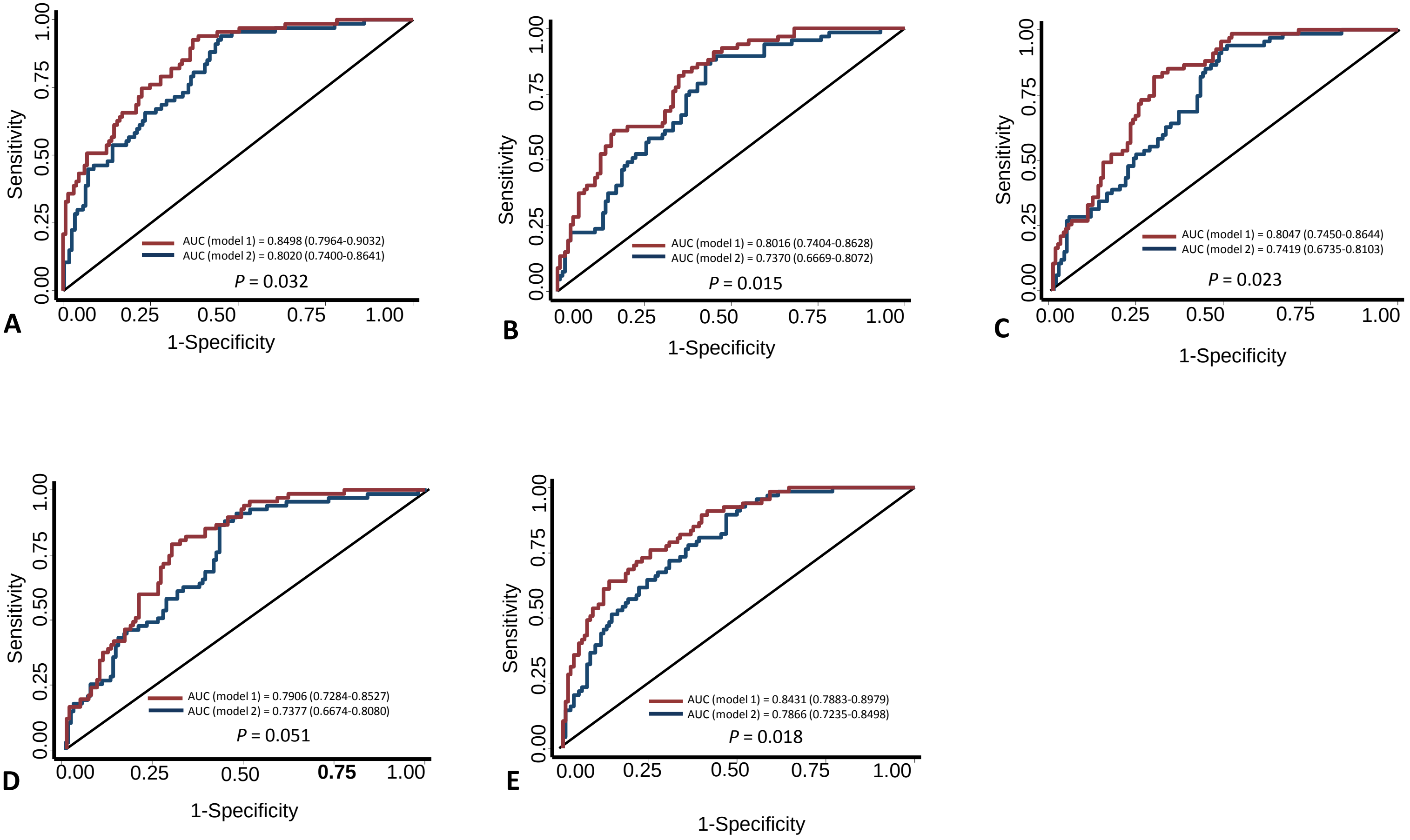

Supplementary Figure 2. Discrimination accuracy for different HCC diagnosis models. Discrimination accuracy for HCC diagnosis was evaluated by AUC of ROC curves. The AUCs were compared between the model including age, gender, smoking status, drinking status, family history of cancer, cirrhosis, and AFP (**A**), or each of the liver enzymes including ALP (**B**), AST (**C**), ALT (**D**), and GGT (**E**), plus mtDNA content (model 1), and the model including the above variables excluding mtDNA content (model 2).
